# Cold Atmospheric Plasma Treatment Induces Anti-Proliferative Effects in Prostate Cancer Cells by Redox and Apoptotic Signaling Pathways

**DOI:** 10.1371/journal.pone.0130350

**Published:** 2015-07-01

**Authors:** Martin Weiss, Denis Gümbel, Eva-Maria Hanschmann, Robert Mandelkow, Nadine Gelbrich, Uwe Zimmermann, Reinhard Walther, Axel Ekkernkamp, Axel Sckell, Axel Kramer, Martin Burchardt, Christopher H. Lillig, Matthias B. Stope

**Affiliations:** 1 Department of Urology, University Medicine Greifswald, Greifswald, Germany; 2 Department of Trauma and Reconstructive Surgery, University Medicine Greifswald, Greifswald, Germany; 3 Department of Medical Biochemistry and Molecular Biology, University Medicine Greifswald, Greifswald, Germany; 4 Department of Hygiene and Environmental Medicine, University Medicine Greifswald, Greifswald, Germany; Wayne State University School of Medicine, UNITED STATES

## Abstract

One of the promising possibilities of the clinical application of cold plasma, so-called cold atmospheric plasma (CAP), is its application on malignant cells and cancer tissue using its anti-neoplastic effects, primarily through the delivery of reactive oxygen and nitrogen species (ROS, RNS). In this study, we investigated the impact of CAP on cellular proliferation and consecutive molecular response mechanisms in established prostate cancer (PC) cell lines. PC cells showed a significantly reduced cell growth following CAP treatment as a result of both an immediate increase of intracellular peroxide levels and through the induction of apoptosis indicated by annexin V assay, TUNEL assay, and the evaluation of changes in nuclear morphology. Notably, co-administration of N-acetylcysteine (NAC) completely neutralized CAP effects by NAC uptake and rapid conversion to glutathione (GSH). Vitamin C could not counteract the CAP induced effects on cell growth. In summary, relatively short treatments with CAP of 10 seconds were sufficient to induce a significant inhibition of cancer proliferation, as observed for the first time in urogenital cancer. Therefore, it is important to understand the mode of CAP related cell death and clarify and optimize CAP as cancer therapy. Increased levels of peroxides can alter redox-regulated signaling pathways and can lead to growth arrest and apoptosis. We assume that the general intracellular redox homeostasis, especially the levels of cellular GSH and peroxidases such as peroxiredoxins affect the outcome of the CAP treatment.

## Introduction

Despite the development of new promising therapeutic strategies against early and advanced urogenital tumors such as prostate, renal or urethral cancer, radical surgery remains the standard therapy as a curative approach. Primarily, prostate cancer (PC) represents one of the most diagnosed malignant diseases and remains second-leading cause of tumor-associated deaths in male in the Western hemisphere [1]. In most fields of surgical oncology there is broad consensus about excision of a tumor in total and with a sufficiently large surgical margin (R0-resection). Surprisingly, clinical trials show that cancer-positive surgical margins are inevitable in a substantial number of cases [2, 3, 4]. However, preferably complete local excision of malignant cells increases the risk of damaging flanking tissues and organs. Therefore, new therapeutic applications are required to prevent cancer-positive surgical margins by eliminating microscopic residues after resection of urogenital tumors, and simultaneously enable to reduce the minimum distance between treated tumor and surrounding tissue.

Recently, cold atmospheric plasma (CAP) indicated promising anti-neoplastic effects on several tumors, e.g. melanoma, glioma, and pancreatic cancer cells [5, 6, 7], and therefore could be an efficient method for anti-cancer treatment in clinical urology in the future. Physical plasma is defined as a highly reactive ionized gas containing diverse biologically reactive factors involving charged particles (ions, electrons), excited atoms and molecules (i.e reactive oxygen and nitrogen species, ROS, RNS), free radicals (atoms or molecules containing an unpaired electron), photons and electromagnetic fields, leading to the emission of visible ultraviolet, vacuum-ultraviolet as well as infra-red radiation [8, 9]. The temperature can be adjusted to body temperature. The reactive compounds, which become biochemically active, emerge during the generation of the plasma by interaction with molecules of the surrounding air, and/or by contact of plasma with either the medium, the bodily fluid, or the tissue to be treated [10, 11, 12]. Treatment of biological tissues and cells with CAP becomes feasible, due to electrons heating up much faster in an electric field compared to ions, resulting in an ambient temperature plasma jet [13]. Clarifying the underlying biological effects and mechanisms of action still remains a considerable challenge, however, there is mounting evidence reporting that ROS are primarily responsible for CAP-dependent cell death [14]. Cancer cell growth is frequently associated with a disruption of physiological redox homeostasis and signaling, for instance, increased levels of 8-hydroxy desoxyguanosin and hydrogen peroxide have been shown in various carcinoma cells [15]. Redox signaling is mediated by members of the thioredoxin family (e.g. thioredoxins, Trxs; glutaredoxins, Grxs; peroxiredoxins, Prxs) that control crucial cellular processes including proliferation and apoptosis. Trxs and Grxs regulate protein function via the reversible reduction/oxidation of specific cysteinyl residues and control intracellular hydrogen peroxide levels by reducing Prxs, which in turn reduce cellular peroxides to water. Thioredoxin isoforms are ubiquitously expressed in mammals, are localized throughout the compartmentalized cells, and show distinct changes in expression in various pathologies including cancer (for an overview see Hanschmann et al. [16]). A strong and sustained disruption of redox signaling, as it could result from CAP exposure, may lead to inhibition of disturbed or disrupted functions of proteins and essential cellular signaling pathways [17].

In this study we describe the effects of CAP treatment on human PC cell lines. Our study does not only support previous data on CAP-driven alteration of ROS, but moreover confirms the specific induction of peroxides, that immediately alter the redox state of Prxs reversibly and potentially other proteins, that promote cell growth arrest and apoptosis, respectively.

## Materials and Methods

### Cell culture

Human epithelial PC cell lines LNCaP and PC-3 (Cell Lines Service, Eppelheim, Germany) were propagated in RPMI 1640 medium supplemented with 10% fetal calf serum and 100 units/ml penicillin/streptomycin (PAN Biotech) at 37°C and 5% CO_2_ in a humidified atmosphere. For cell transfer onto a cell culture plate adherent cells were detached by 0.1% trypsin/0.04% ethylenediaminetetraacetic acid (EDTA) and resuspended in RPMI medium. For further experiments RPMI medium was supplemented with 5 mM N-acetylcysteine (NAC; Carl Roth, Karlsruhe, Germany), 100 μM vitamin C (Carl Roth) or 5 μg/ml cycloheximide (Merck, Darmstadt, Germany), respectively.

### CAP treatment

**Fig 1:**
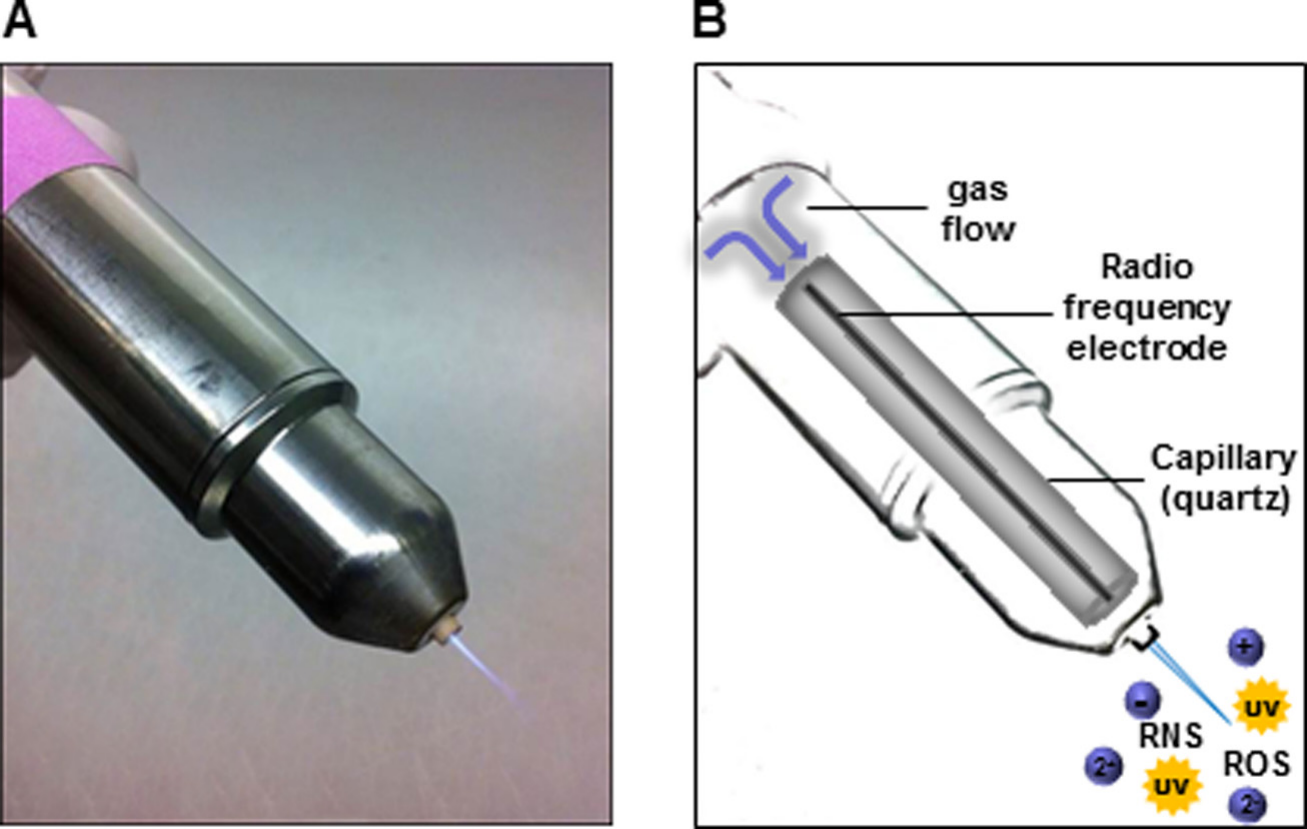
The cold atmospheric plasma (CAP) source. (A) Plasma jet kINPen09 (Neoplas GmbH, Greifswald, Germany), (B) schematic reconstruction of kINPen09 and composition of physical plasma.

For generating CAP with argon as a carrier gas, the atmospheric pressure plasma jet kINPenMed (Neoplas GmbH, Greifswald, Germany) was utilized (Fig 1). Suspended cells were treated in 500 μl RPMI 1640 medium (PAN Biotech, Aidenbach, Germany) on an uncoated cell culture plate. The major reason for cell treatment in suspension was to avoid any side effects from the CAP source when treating adherent cells, i.e. mechanical damage and drying. Proliferation assays were propagated with 3x10^4^ LNCaP and PC-3 cells. Further assays and Western Blot analysis were performed with 6x10^5^ LNCaP and PC-3 cells The CAP source was applied for 10 s and controls were treated with non-ionized argon gas for 10 s, respectively. Following CAP treatment cells were immediately transferred to poly-L-lysine (Sigma-Aldrich, St. Louis, USA) coated cell culture plates and were cultured in RPMI 1640 medium for indicated time points. For CAP treatment with the kINPen09 the following setting was used: Argon gas flow: 3 l/min; supply voltage = 65 V DC; frequency: 1.1 MHz; exposure time: 10 s. Control cells: Argon gas flow: 3 l/min; exposure time: 10 s [18, 7].

### Proliferation assay

Cellular proliferation of LNCaP and PC-3 cells was analysed in 24-well cell culture plates (1 ml/well) using a CASY Cell Counter and Analyzer Model TT (Roche Applied Science, Mannheim, Germany) over a period of 120 h. Adherent cells were detached by 0.1% trypsin/0.04% ethylenediaminetetraacetic acid (EDTA) and resuspended in CASYton solution (Roche Applied Science). The number of living cells was determined in duplicates for each sample.

### Annexin V assay

LNCaP and PC-3 cells were suspended in 500 μl RPMI media and were CAP treated for 10 sec. Argon treated cells served as control. After 4 h of incubation adherent cells were detached by 0.1% trypsin/0.04% EDTA, washed with PBS and resuspended in binding buffer. AnnnexinV assay was performed using the FITC Annexin V Apoptosis Detection Kit I (BD Bioscience, Germany) as recommended by the supplier.

### Terminal deoxynucleotidyl transferase dUTP nick end labeling (TUNEL) assay

6x10^5^ suspended LNCaP and PC-3 cells were suspended in 500 μl RPMI media and CAP treated for 10 sec. Argon treated cells served as control. After 24 h of incubation adherent cells were detached by 0.1% trypsin/0.04% EDTA. TUNEL apoptosis analysis was performed using the HT TiterTACS Assay Kit (Trevigen, Gaithersburg, USA) following supplier recommendations. Data were acquired using an Infinite 200 PRO multimode reader (Tecan) and were analyzed using the i-control 1.9 software (Tecan).

### Nuclear morphology assay

6x10^5^ suspended LNCaP and PC-3 cells were CAP treated for 10 sec and seeded onto a 6-well cell culture plate containing 2.5 ml RPMI medium. Argon treated cells served as control. Cells were cultured for 24 h, were washed with PBS, fixed with 4% paraformaldehyde for 15 min, rinsed twice with PBS and incubated with 0.2% Triton-X-100 for 5 min. Cells were washed with PBS and stained with 4′,6-diamidino-2-phenylindole (DAPI, Carl Roth) for fluorescence analysis using a BZ-9000 fluorescent microscope system (Keyence, Osaka, Japan) and a BZ II Analyzer software (Keyence). During apoptosis cells underlie nuclear morphology changes, chromatin condensation, and degradation into nuclear body fragments [19, 20, 21]. For detection of morphological changes fluorescent microscopic images were taken from 10 positions per 3.5-cm-cell culture dish, representing 0.5% of the total growth area. Computational morphometric analysis was performed for each single nucleus. Total numbers of DAPI positive nuclei ranged from 400–600 (CAP / cycloheximide treated cells) and 700–1000 (argon / vehicle treated cells). The morphometric parameters were calculated by the BZ II Analyzer software. Therefore, the Hybrid Cell Count BZ-H2C module was used to define the parameters of selection. Single nuclei were defined by Colocalization function as DAPI positive areas in a range from 0.3 to 200 μm^2^, excluding nuclei cluster and fragmented nuclei. The morphometric parameters were obtained by the software analysis tools area, perimeter, major axis, minor axis, and brightness. Apoptotic condensation represented by nuclei shrinkage is characterized by a decrease of nuclear area and perimeter. Further morphological chances accompanied with nuclear deformation and disrounding constitute disproportions of the parameters major and minor axis, as well as an increase of nuclear brightness.

### Western blotting

#### 2-Cys Prxs redox blot

To determine the CAP-induced changes in the cellular levels and the redox state of Prx1, Prx2 and Prx3 6x10^5^ suspended LNCaP and PC-3 cells were treated with CAP or argon for 10 s. Cells were centrifuged at 1000 rpm for 5 min. The supernatant was collected and mixed with loading dye (0.3 M Tris/HCl (pH 7.0), 50% glycerol, 5% SDS, 0.1% bromphenol blue) and was frozen at -20°C for later analysis of potential secretion of Prx1 and Prx2. Cells were incubated for 15 min in PBS containing 100 mM of the alkylating agent N-ethylmaleimide (NEM) that irreversibly binds to free thiol groups. After centrifugation at 1000 rpm for 5 min, the supernatant was discarded and the pellet was resuspended in NEM-containing buffer (40 mM HEPES (pH 7.4), 50 mM NaCl, 1 mM EDTA, 1 mM EGTA, protease inhibitors, 100 mM NEM) for 15 min and lysed by the addition of 2% CHAPS. Total protein amount was determined according to Bradford and 10–20 μg of protein were diluted and mixed with loading dye. SDS PAGE was run using pre-casted gels (4–20%, BioRad) and the stain-free technology (BioRad). To analyse the redox state of the cellular Prxs, proteins analysed by non-reducing SDS PAGE. In order to analyse secreted Prxs in the supernatant, equal volumes were reduced with 100 mM DTT for 30 min at room temperature and 10 min at 94°C. Proteins were separated by SDS PAGE and were transferred to PVDF membranes using the semi-dry technique. Membranes were blocked with 5% milk powder and 1% BSA in TBS containing 0.05% Tween-20. Prx1, Prx2 and Prx3 were analysed using specific primary antibodies (produced and evaluated as stated in [22] and [23]), a HRP-labeled secondary antibody and the enhanced chemoluminescence development technique using the ChemiDoc system (BioRad). Levels of oxidized Prxs were quantified by densitometric analysis using ImageJ and the ImageLab software (BioRad) and were normalized to the total amount of the particular Prx. Moreover, total protein was quantified based on the stain-free technology and was used for normalization of the blotting data obtained from densitometric analysis.

### Glutathione (GSH) assay

LNCaP and PC-3 cells were incubated in presence or absence of 5 mM NAC over a period of 12 h. Cells were harvested by 0.1% trypsin/0.04% EDTA, washed with PBS and lysed in lysis buffer (40 mM HEPES (pH 7.4), 50 mM NaCl, 1 mM EDTA, 1 mM EGTA, protease inhibitors, and 2% CHAPS). The protein amount was determined according to Bradford. 100 μg of protein were precipitated over night using 4% sulfosalicylic acid. Samples were centrifuged at 4°C for 30 min at 13000 rpm. Supernatants were neutralized using NaOH and were analysed for total GSH content in a colorimetric assay against a GSH standard with known concentrations (0–0.2 mM). The assay is based on the formation of the yellow TNB from 5,5′-Dithio-bis-(2-Nitrobenzoic acid) (DTNB) in the presence of GSH. The assay was performed in a 96 well plate; the reaction mixture contained 1.5 mM NADPH (Carl-Roth), yeast GSH reductase and 1.5 mM DTNB (Carl-Roth) and was measured at 412 nm as end-point assay using the Infinite 200 PRO multimode reader (Tecan).

### Statistics

Statistical comparisons were performed from at least 9 independent growth kinetic experiments and in case of other assays from a minimum of 3 independent experiments using the unpaired Student’s *t* test. Results of *p*<0.05 were considered significant. Data are given as mean ± SD.

## Results

### CAP treatment inhibited cellular proliferation of human PC cell lines

**Fig 2:**
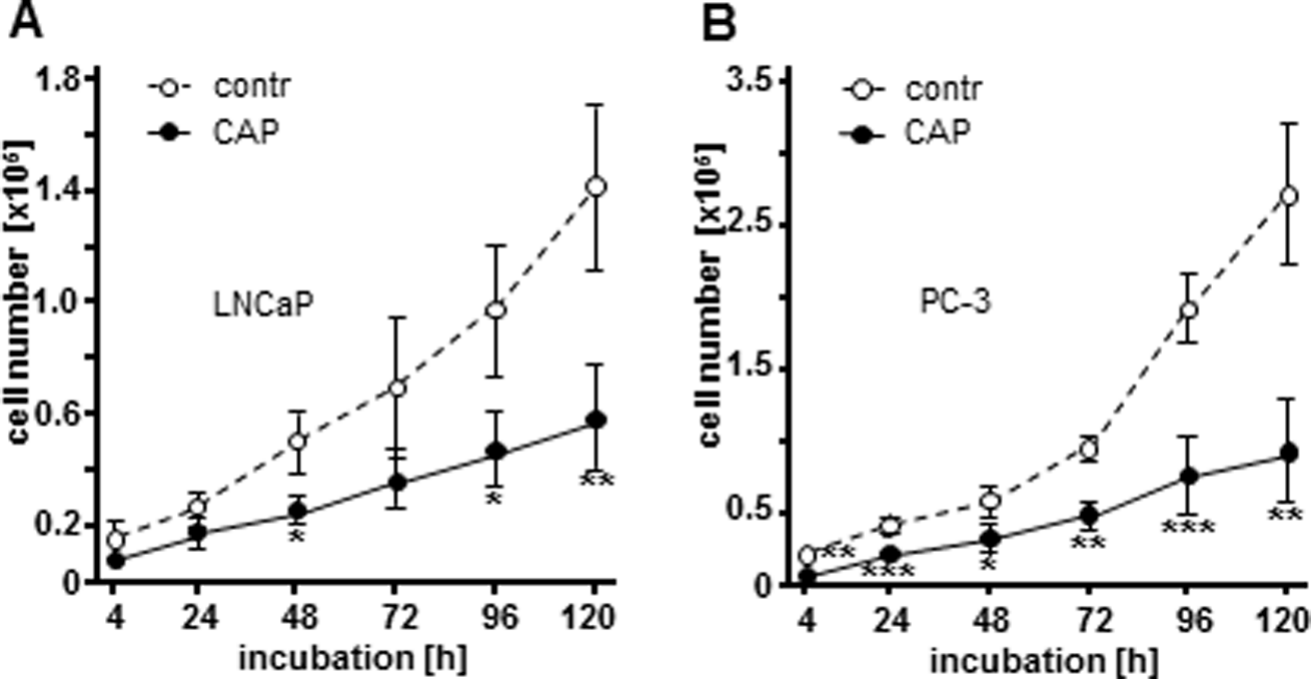
Cold atmospheric plasma (CAP) inhibits cellular growth of human prostate cancer cells. Following CAP treatment for 10 s LNCaP and PC-3 cells were counted using a CASY Cell Counter and Analyzer Modell TT (Roche Applied Science) at indicated time points following. Control cells were treated for 10 s with argon (contr). Living cell number of (A) LNCaP cells, and (B) PC-3 cells treated with CAP revealed significantly decreased cell numbers compared to controls. (C) LNCaP cells incubated with 10 nM docetaxel showed significantly inhibited cell growth, comparable to CAP treatment. Results are expressed as the mean ± SD of cell count. *p<0.05; ***p<0.001, as determined by Student’s *t* test.

In this study we aimed at analysing the short- and long-term effects of CAP on proliferation of human PC cell lines LNCaP and PC-3 after a single CAP treatment of 10 s. Argon treated cells were used as control. Using a CASY Cell Counter and Analyser Model TT the total cell number was analysed for a period of 120 h (Fig 2A and 2B). 10 s CAP application caused significant reductions in the total cell numbers of the observed cell lines LNCaP (Fig 2A; 4 h: 2.15 fold, *p* = 0.1138; 24 h: 1.36 fold, *p* = 0.2034; 48 h: 1.79 fold, *p* = 0.0227; 72 h: 1.88 fold, *p* = 0.0584; 96 h: 1.96 fold, *p* = 0.0148; 120 h: 2.30 fold, *p* = 0.0050) and PC-3 (Fig 2B; 4 h: 3.23 fold, *p* = 0.0040; 24 h: 1.82 fold, *p* = 0.0007; 48 h: 1.90 fold, *p* = 0.0177; 72 h: 2.01 fold, *p* = 0.0011; 96 h: 2.58 fold, *p* = 0.0010; 120 h: 2.96 fold, *p* = 0.0015) compared to argon-treated controls.

### CAP-induced apoptosis in LNCaP and PC-3 cells

**Fig 3:**
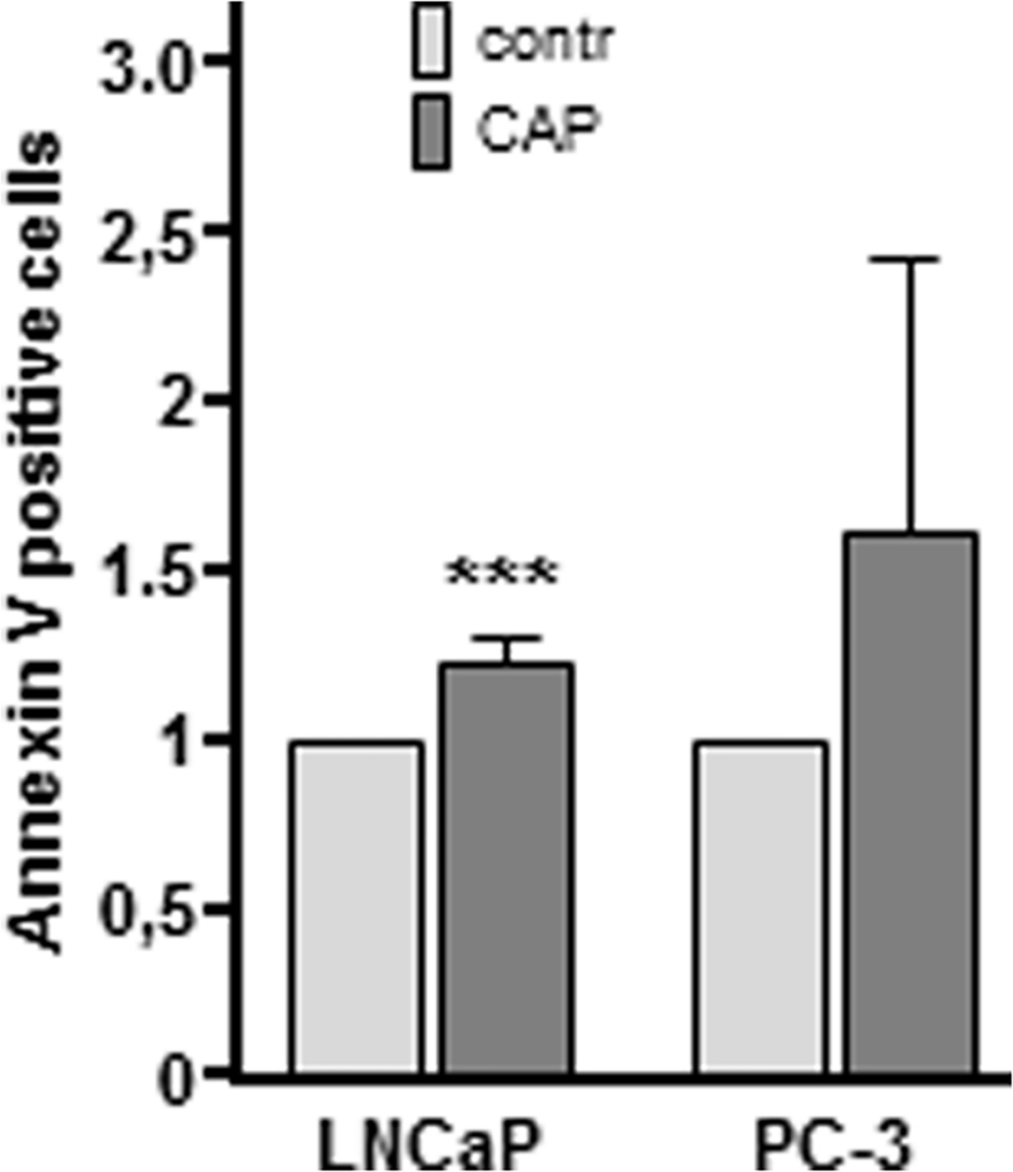
Increased detection of membranous phosphatidylserine by Annexin V following cold atmospheric plasma (CAP) exposure. LNCaP and PC-3 cells were treated with CAP for 10 s and were cultured for 24 h. Argon treated cells were used as control (contr). Cells were harvested and processed using a FITC Annexin V Apoptosis Detection Kit I (BD Bioscience, Germany) according to manufactures' instructions. 24 h after CAP treatment LNCaP and PC-3 cells showed an increase of phosphatidylserine-bound Annexin V. Data are expressed as the mean ±SD. ***p<0.01, as determined by Student’s *t* test.

To proof whether the reduction of cell numbers after CAP treatment is the result of apoptotic cell death, we further examined the effects of CAP treatment by annexin V and TUNEL assay as well as evaluation of nuclear morphology by the nuclear morphology assay. Staining of membranous phosphatidylserine via annexin V, i.e. among others, a crucial factor for apoptosis when being externalized to the outer plasma membrane, showed an accumulation of annexin V signal 4 h after CAP treatment of LNCaP and PC-3 cells (Fig 3; LNCaP: 1.25 fold, *p* = 0.0007; PC-3: 1.60 fold, *p* = 0.2954). The results were significant for LNCaP, whereas despite a respectable increase of annexin V signal, not significant for PC-3. For this reason we enforced apoptosis detection by TUNEL assay and nuclear morphology assay to visualize changes in nuclear morphology.

**Fig 4:**
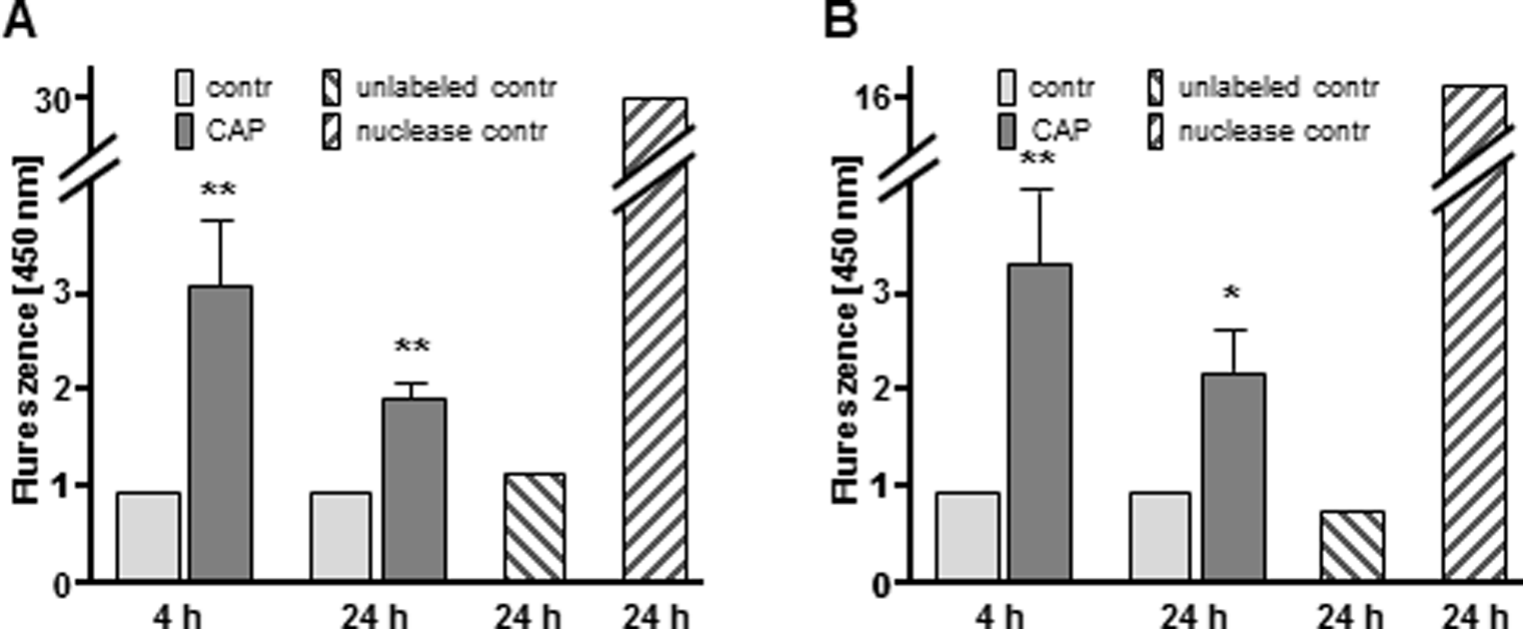
Changes in LNCaP and PC-3 nuclei following cold atmospheric plasma (CAP) exposure. LNCaP and PC-3 cells were treated with CAP for 10 s and were cultured for 4 h or 24 h. Argon treated cells were used as control (contr). Cells were harvested and processed by specific labeling of nuclear DNA fragmentation with a HT TiterTACS Assay Kit (Trevigen, Gaithersburg, USA) according to manufactures' instructions. (A) Nuclear DNA fragmentation of LNCaP and (B) PC-3 cells after 10 sec of CAP treatment (CAP) compared to argon treated controls (contr). Unlabeled control cells (unlabeled contr) served as negative control and nuclease treated cells (nuclease contr) served as positive control, respectively. Data are expressed as the mean ±SD. *p<0.05, **p<0.01, as determined by Student’s *t* test.

**Fig 5:**
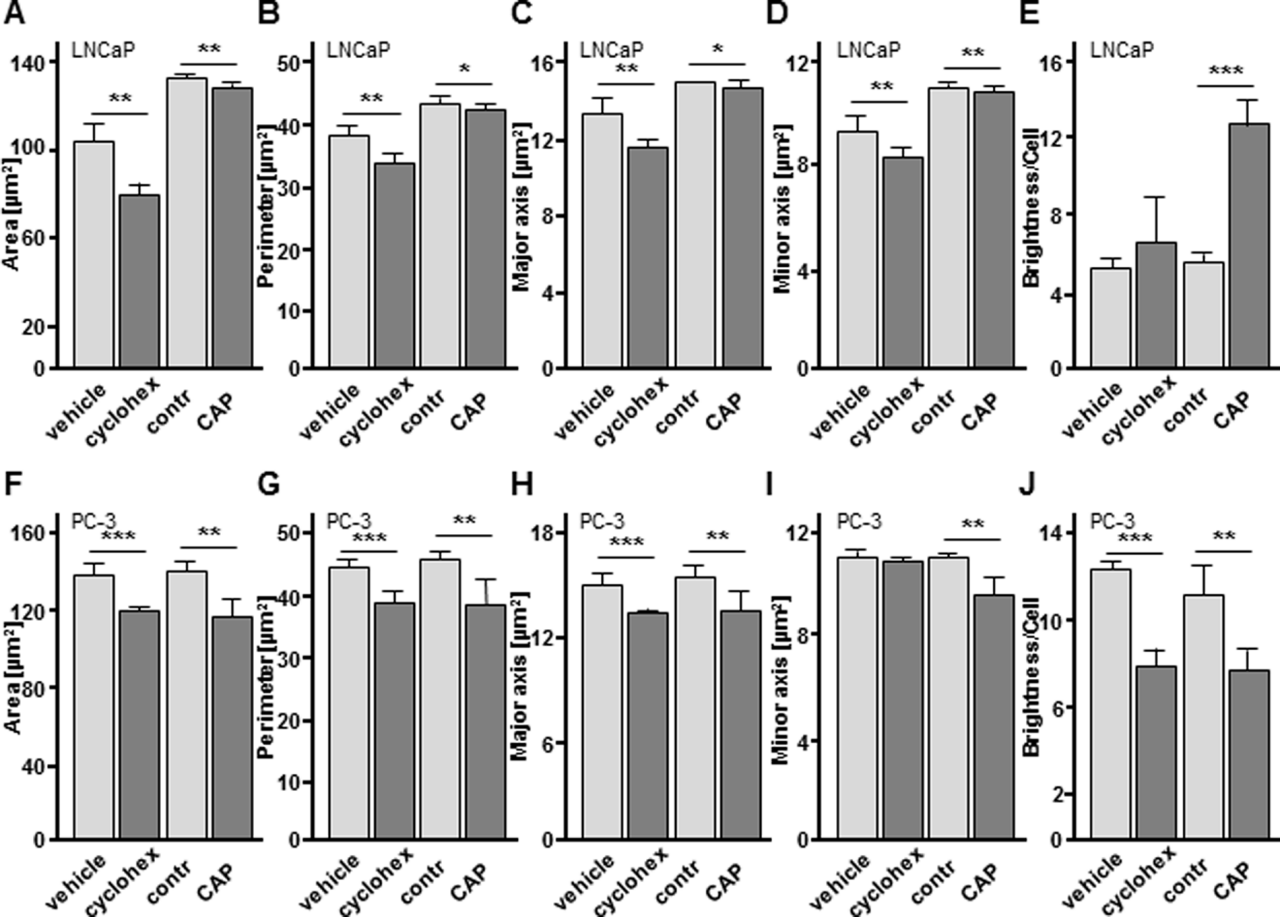
Cold atmospheric plasma (CAP) treatment causes morphological changes to DNA and nucleus visualized by nuclear morphology assay. LNCaP and PC-3 cells were CAP treated for 10 sec, fixed with 4% paraformaldehyde for 15 min and stained with 4′,6-diamidino-2-phenylindole (DAPI) for fluorescence analysis. Nuclear parameters area (A, F), perimeter (B,G), major axis (C,H), minor axis (D,I) and brightness per cell (E,J) after DAPI fluorescence of LNCaP and PC-3 cells after CAP and argon (contr) treatment. Incubation with cycloheximide (cyclohex) and its vehicle served as internal positive control for apoptosis. Data are expressed as the mean ±SD. *p<0.05, **p<0.01, ***p<0.001, as determined by Student’s *t* test.

By labeling DNA fragments through TUNEL assay both LNCaP and PC-3 cells showed a significantly higher severity of DNA fragmentation and therefore an increase in TUNEL positive cell area after CAP treatment compared to argon treated controls (Fig 4A; LNCaP; 4 h: 3.07 fold, *p* = 0.0068; 24 h: 1.93 fold, *p* = 0.0012; Fig 3B; PC-3; 4 h: 3.35 fold, *p* = 0.0078; 24 h: 2.24 fold, *p* = 0.0384). Moreover, the nuclear morphology assay is in wide use for detecting apoptosis in eukaryotic cells and other cell types, as it is an indicator for morphologic changes of the nucleus. As depicted in Fig 5, the nuclear morphology assay of CAP treated LNCaP and PC-3 cells revealed significant and apoptosis-associated alterations of nuclear morphology concerning area (Fig 5A; LNCaP: 1.04 fold decrease, *p* = 0.0022; Fig 5F; PC-3: 1.27 fold decrease, *p* = 0.0021), perimeter (Fig 5B; LNCaP: 1.02 fold decrease, *p* = 0.0310; Fig 5G; PC-3: 1.11 fold decrease, *p* = 0.0065), major axis (Fig 5C; LNCaP: 1.02 fold decrease, *p* = 0.0405; Fig 5H; PC-3: 1.13 fold decrease, *p* = 0.0050) and minor axis (Fig 5D; LNCaP: 1.03 fold decrease, *p* = 0.0085; Fig 5I; PC-3: 1.14 fold decrease, *p* = 0.0051), as well as brightness per cell (Fig 5E; LNCaP: 2.25 fold increase, *p*<0.0001; Fig 5J; PC-3: 1.45 fold decrease, *p* = 0.0034), compared to argon treated controls. The induction of apoptosis by cycloheximide incubation served as internal control [24] and caused comparable alterations of area (Fig 5A; LNCaP: 1.27 fold decrease, *p* = 0.0028; Fig 5F; PC-3: 1.14 fold decrease, *p* = 0.0006), perimeter (Fig 5B; LNCaP: 1.13 fold decrease, *p* = 0.0071; Fig 5G; PC-3: 1.09 fold decrease, *p* = 0.0005), major axis (Fig 5C; LNCaP: 1.15 fold decrease, *p* = 0.0099; Fig 5H; PC-3: 1.13 fold decrease, *p* = 0.0001) and minor axis (Fig 5D; LNCaP: 1.13 fold decrease, *p* = 0.0012; Fig 5I; PC-3: 1.02 fold decrease, *p* = 0.4306), as well as brightness per cell (Fig 5E; LNCaP: 1.23 fold increase, *p*<0.0001; Fig 5J; PC-3: 1.55 fold decrease, *p* = 0.0002).

### Incubation with NAC reversed anti-proliferative CAP effects

**Fig 6:**
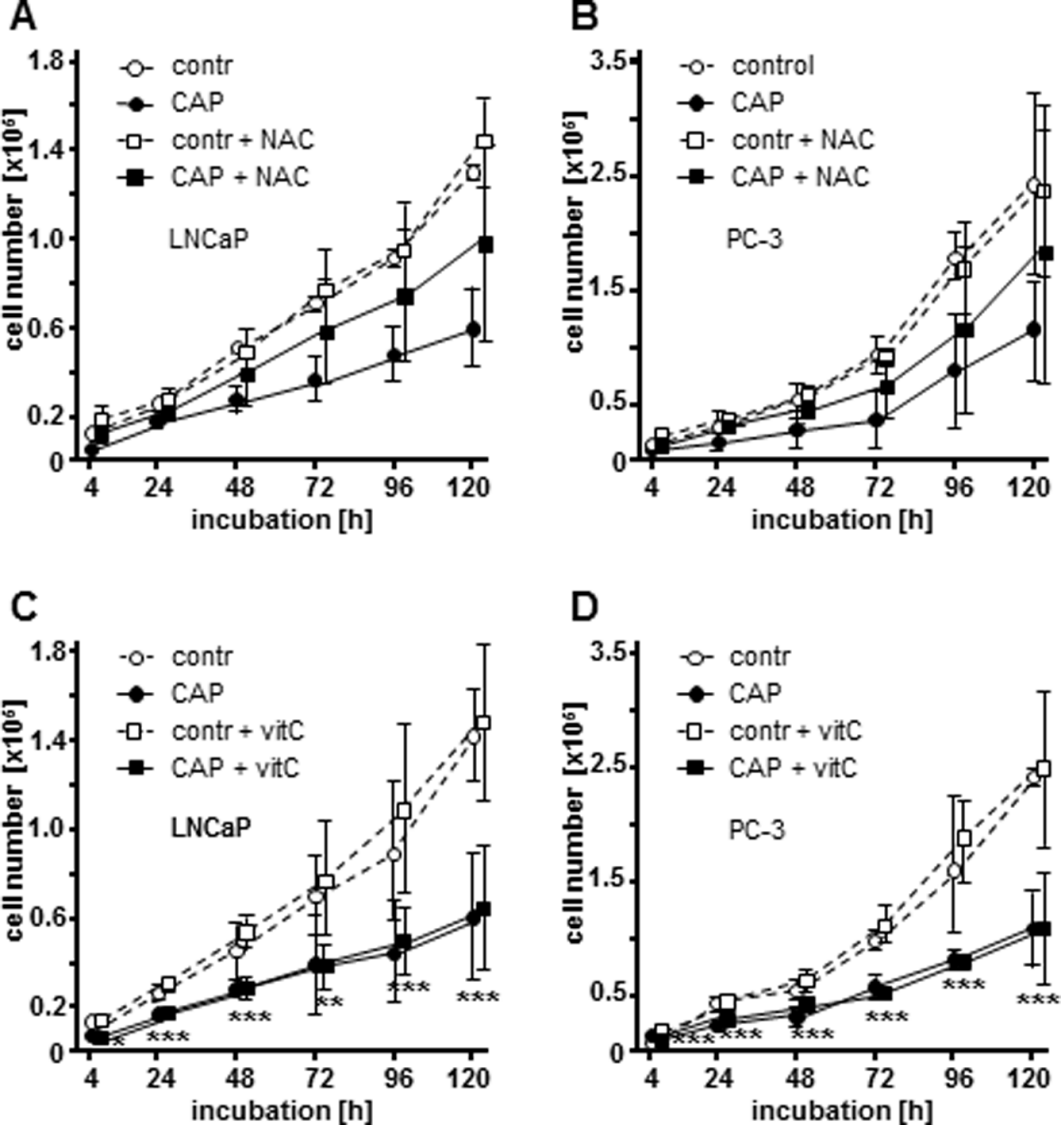
N-acetylcysteine (NAC) reverses anti-proliferative effects of cold atmospheric plasma (CAP) on LNCaP and PC-3 cells. Living cell number of LNCaP and PC-3 cells after CAP treatment for 10 s. CAP-treated cells were incubated with cell culture medium in the absence of NAC and vitamin C (○ and ●), (A+B) cell culture medium containing 5 mM NAC (□ and ■), and (C+D) cell culture medium containing 100 μM vitamin C (VitC, □ and ■) over a period of 120 h. Cells were counted using a CASY Cell Counter and Analyzer Modell TT (Roche Applied Science) at indicated time points. Results are expressed as the mean ±SD of cell count. *p<0.05, **p<0.01, ***p<0.001, as determined by Student’s *t* test.

Recently, there is growing evidence that anti-proliferative CAP effects on mammalian cells are caused by the induction of reactive species including ROS and RNS [4]. To determine whether reactive species are responsible for the decrease of cellular growth in PC, we counted living cells of CAP or argon treated LNCaP and PC-3 cells that were incubated in the presence or absence of NAC and vitamin C for up to 120 h (Fig 6). Both reagents are known to mediate universal antioxidant effects [22]. Again, the application of CAP on cells grown in RPMI cell culture medium revealed significantly decreased cell numbers of LNCaP (Fig 6A; 4 h: 2.04 fold, *p* = 0.0328; 24 h: 1.30 fold, *p* = 0.1277; 48 h: 1.58 fold, *p* = 0.0298; 72 h: 1.77 fold, *p* = 0.0275; 96 h: 2.00 fold, *p* = 0.0189; 120 h: 2.30 fold, *p* = 0.0205) as well as PC-3 cells (Fig 6B; 4 h: 2.63 fold, *p* = 0.0005; 24 h: 2.08 fold, *p* = 0.0008; 48 h: 2.07 fold, *p* = 0.0044; 72 h: 2.61 fold, *p* = 0.0048; 96 h: 2.29 fold, *p* = 0.0509; 120 h: 2.12 fold, *p* = 0.0056) compared to argon treated controls and was comparable to what we have shown before (Fig 2). Notably, the supplementation of NAC to CAP treated LNCaP and PC-3 cells largely removed the growth inhibiting effect of CAP as NAC-incubated cell lines exhibited increased cell growth after plasma treatment compared to CAP treated cells in the absence of NAC (Fig 6A; LNCaP; 4 h: 2.30 fold, *p* = 0.1084; 24 h: 1.27 fold, *p* = 0.1474; 48 h: 1.38 fold, *p* = 0.1853; 72 h: 1.50 fold, *p* = 0.0952; 96 h: 1.52 fold, *p* = 0.1323; 120 h: 1.60 fold, *p* = 0.0134; Fig 6B; PC-3; 4 h: 1.95 fold, *p* = 0.0287; 24 h: 1.81 fold, *p* = 0.1111; 48 h: 1.61 fold, *p* = 0.1011; 72 h: 1.73 fold, *p* = 0.0571; 96 h: 1.43 fold, *p* = 0.0917; 120 h: 1.57 fold, *p* = 0.2247). Most interestingly, the antiproliferative CAP effects were not limited by the antioxidant vitamin C as the proliferation of CAP treated cells in the presence or absence of vitamin C statistically not differed. CAP treated cells supplemented with vitamin C demonstrated a statistically significant reduction of LNCaP and PC-3 proliferation compared to argon treated controls (LNCaP; Fig 6C; 4 h: 1.85 fold, *p* = 0.0397; 24 h: 1.61 fold, *p*<0.0001; 48 h: 1.76 fold, *p*<0.0001; 72 h: 1.91 fold, *p* = 0.0020; 96 h: 2.16 fold, *p* = 0.0007; 120 h: 2.20 fold, *p* = 0.0001; PC-3; Fig 6D; 4 h: 2.24 fold, *p*<0.0001; 24 h: 1.80 fold, *p*<0.0001; 48 h: 1.74 fold, *p*<0.0001; 72 h: 2.25 fold, *p*<0.0001; 96 h: 2.35 fold, *p*<0.0001; 120 h: 2.30 fold, *p* = 0.0002).

**Fig 7:**
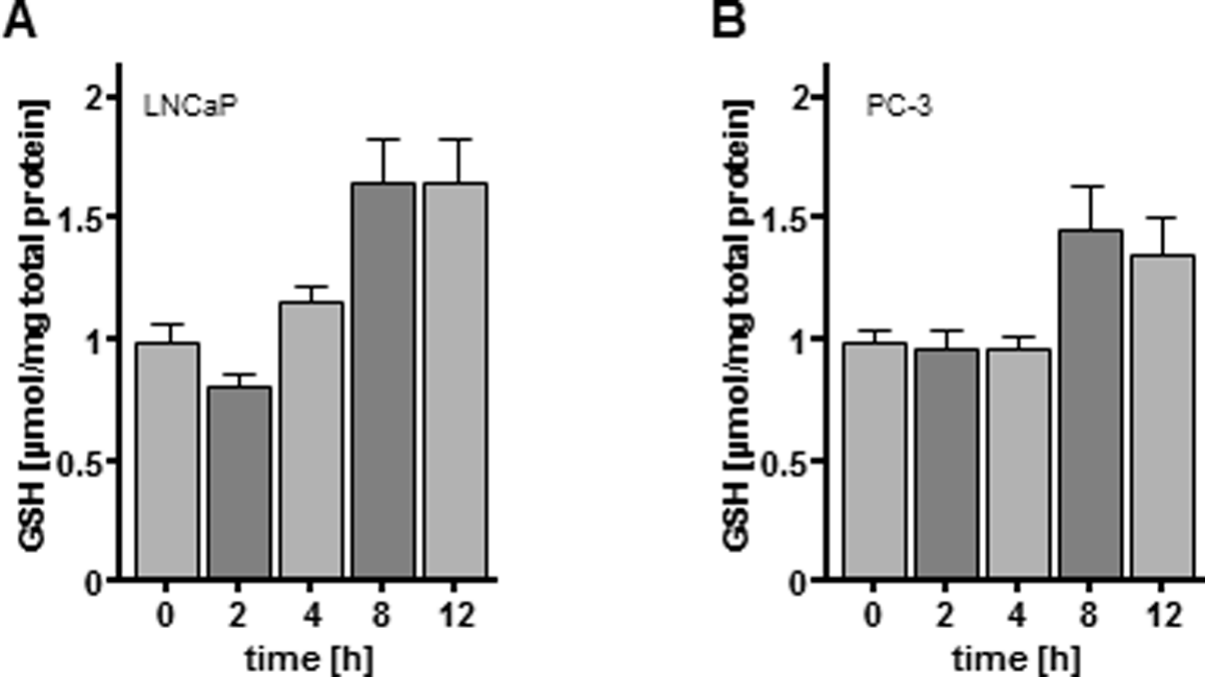
Intracellular glutathione (GSH) levels following N-acetylcysteine (NAC) treatment. LNCaP and PC-3 cells were treated with 5 mM NAC for a period of 12 h. Cells were harvested and lysed at different time points. Total GSH levels were analysed in a colorimetric assay using 5,5′-Dithio-bis-(2-Nitrobenzoic acid) (DTNB) as substrate. GSH levels were quantified against GSH standards and are depicted as μmol/mg total protein. 4 h (LNCaP) and 12 h (PC-3) following NAC treatment a significant increase in total GSH levels was detected. Results are expressed as the mean ±SD of cell count.

NAC is an established source for GSH synthesis and thus a source for the most abundant two-electron donor, while vitamin C is a more efficient one-electron donor. Next, we evaluated whether the protective effects of NAC treatment could be based on increased intracellular GSH levels using a colorimetric assay for total cellular GSH (Fig 7). In fact, NAC treatment led to an increase in intracellular GSH levels within only a few hours post treatment (LNCaP; Fig 7A; 4 h: 1.18 fold; 8 h: 1.66 fold; 12 h: 1.66 fold; PC-3; Fig 7B; 8 h: 1.47 fold; 12 h: 1.37 fold).

**Fig 8:**
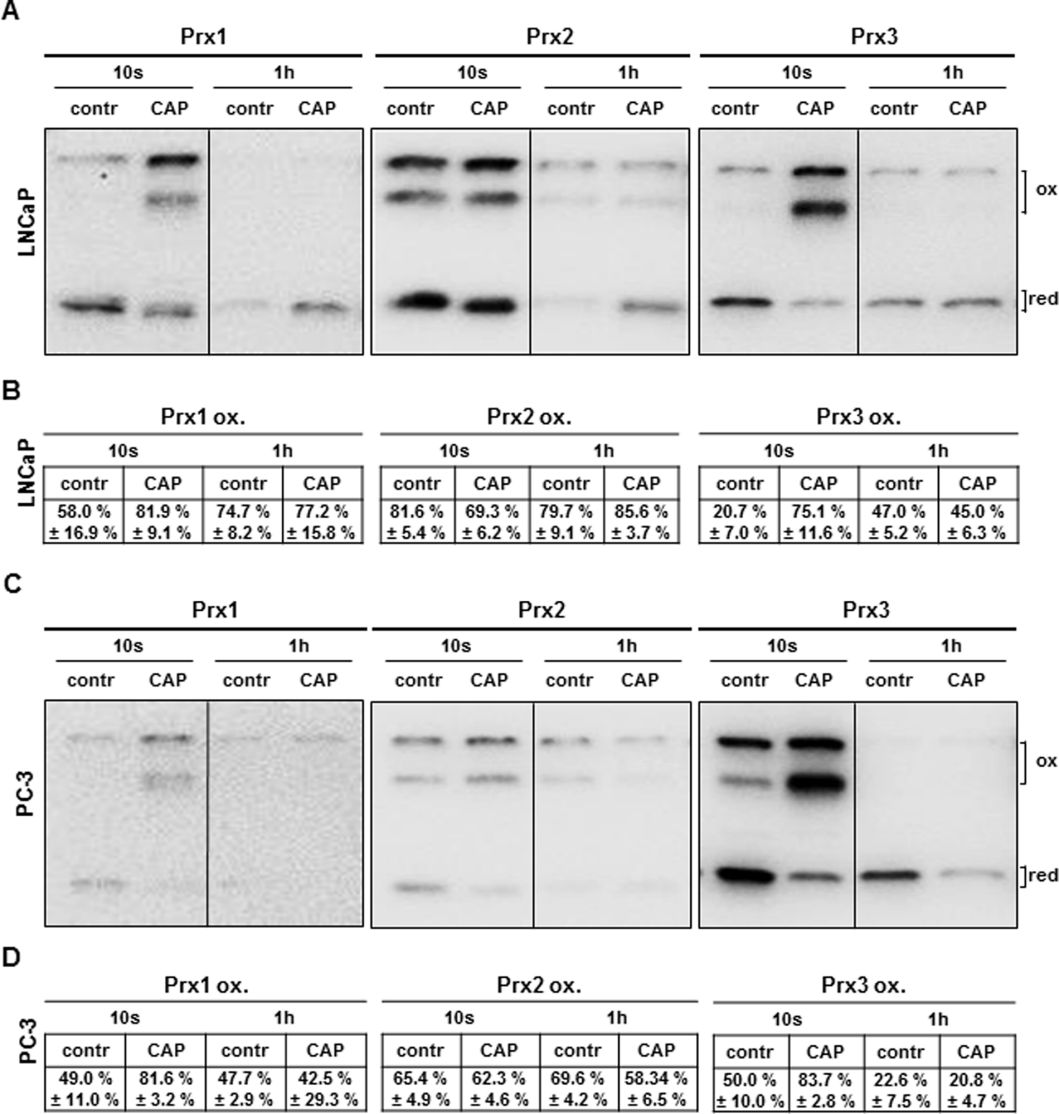
Cold atmospheric plasma (CAP) affects cellular peroxiredoxins (Prxs). LNCaP and PC-3 cells were treated for 10 s with CAP or argon (contr), respectively. Immediately as well as 1 h after the treatment, cells were treated with the alkylating agent N-ethylmaleimide (NEM) which irreversibly binds to reduced thiol groups prior to cell lysis. The protein levels of cytosolic Prx1 and 2, as well as mitochondrial Prx3 were analysed in cell lysates, as well as the redox state using non reducing SDS Page and Western Blot. A) Representative Prx redox blots of the three analysed peroxidases Prx1, Prx2 and Prx3, showing the monomeric Prx and the during the catalytic mechanism generated oxidized, dimeric form. B) Densitometric quantification of the oxidized Prxs compared to total Prx protein (monomeric and dimeric form).

To analyse whether CAP treatment induces the production of ROS or rather increases the intracellular levels of peroxides, we analysed the protein levels and the redox state of the 2-Cys Prx1, Prx2 and Prx3. Additionally, we investigated a potential secretion of Prx1 and Prx2, which are primarily localized in the cytosol, into the extracellular fluid; Prx3 is exclusively located in mitochondria, and therefore a Prx3 secretion was not expected [16, 17]. During their catalytic mechanism reducing peroxides to water, Prxs form an intermolecular disulfide bond. The shift in size can easily be detected by a Prx redox blot, with cell samples being pre-treated with the alkylating agent NEM, which irreversibly binds reduced thiol groups and protects them from further oxidation. Since ROS production potentially is an immediate cause of the CAP treatment, we harvested LNCaP and PC-3 cells immediately and 1 h after the 10 s CAP treatment. Compared to argon treated controls, CAP treatment exaggerated the secretion of the cytosolic Prx1 to the extracellular fluid after 1 h (data not shown). No significant changes in the secretion of Prx2 could be detected. Moreover we could not detect significant alterations in the protein level of any of the 2-Cys-Prxs (Fig 8). However, for both cell lines LNCaP and PC-3, Prx1 and Prx3 showed an increased amount of the oxidised, dimeric form following immediately after CAP treatment (Fig 8; Prx1; LNCaP: 1.41 fold; PC-3: 1.67 fold; Prx3; LNCaP: 3.63 fold; PC-3: 1.67 fold), measured as the ratio of the oxidised, dimeric protein compared to the total (monomeric plus dimeric) protein upon the CAP treatment, compared to argon-treated control cells. Surprisingly, this immediate oxidation after CAP exposure could not be detected for Prx2. To analyse if the enhanced amount of oxidised Prx proteins is not only a short term effect of CAP treatment, we also analysed LNCaP and PC-3 cells 1 h after the CAP treatment. 1 h after CAP treatment we could not detect any more distinct alteration of the amount of the oxidised, dimeric form of Prx1, Prx2 and Prx3 in both cell lines compared to argon-treated control cells. Again, no significant changes in the expression or secretion of the 2-Cys Prxs were detectable (Fig 8A). Nevertheless, we detected a slightly elevated Prx2 oxidation in LNCaP cells 1 h following CAP treatment using the 2-Cys redox blot (Fig 8).

## Discussion

CAP’s molecular mode of action remains poorly understood. The present study critically examines the impact of CAP on PC cells and aims to investigate the so far unknown molecular mechanisms underlying the CAP treatment. Despite the growing knowledge about the CAP impact on several tumor cells, CAP recently was subject to the caveat whether its anti-proliferative effect on cancer cells occurs by necrosis or apoptosis, which to clarify is even more challenging, as the variety of CAP studies used different plasma devices, parameters and tissues. Here, we demonstrated that CAP significantly attenuates cellular proliferation (Fig 2). This effect has been shown in two distinct PC cell lines, LNCaP and PC-3 (Fig 2), which are both well-established as in vitro models for PC, generally differing by hormone sensitivity (LNCaP) and hormone insensitivity (PC-3). Moreover, we ascertained various morphological and functional changes after CAP treatment. As shown by annexin V assay, TUNEL assay, as well as nuclear morphology assay, our data pointed towards apoptotis being the key factor of CAP induced cell death. Indeed, apoptosis is a multi-stage process, which enables the use of different techniques for apoptosis measurement. Annexin V binds phosphatidylserine with a high affinity, which was demonstrated to be externalized from the inner to the outer plasma membrane surface during initiation of apoptosis [25]. Nevertheless, even surface phosphatidylserine localisation is not exclusively linked with apoptosis as this effect was shown in context of any damage of cell membrane such as cell culture handling [26]. In this study LNCaP and PC-3 cells revealed increased surface phosphatidylserine localisation levels after 4 h following 10 sec CAP treatment (Fig 3), which is relatively late as phosphatidylserine translocation was demonstrated to be a very early marker of apoptosis [27]. Even though lacking statistical significance, our study revealed higher signals for CAP dependent surface phosphatidylserine localisation in PC-3 cells than in LNCaP cells. Both cell lines are widely accepted cell culture models for PC and moreover, are known to exhibit striking differences in their set of molecular factors and regulatory network architecture. Due to severe dysregulation of even apoptosis signaling in PC-3 cells some molecular factors are insensitive to apoptotic pathways, e.g. PARP cleavage (unpuplished data), p53, p21, and Bax compared to LNCaP cells [28]. It could be speculated that these mechanistic differences between the two cell lines may cause discrepancies monitored by the apoptosis assays utilized in our study. However, to definitely verify our assumption of apoptotic events arising after cold atmospheric plasma treatment we utilized two more methodologically sound and commercially available techniques for apoptosis measurement. By TUNEL assay we could demonstrate a significant CAP dependent induction of apoptosis (Fig 4). Even though, genome degradation in both cell lines was highly comparable, however PC-3 cells seemed to be more sensitive to control nuclease digestion.

Due to the fact, that artificial and/or non-apoptotic genomic DNA degradation cannot be definitely excluded [29, 30, 31], we performed a nuclear morphology assay as a third and well accepted method to detect nuclear alterations during apoptosis, based on morphometric analysis of DAPI stained nuclei [32, 33]. A parallel approach with cycloheximide-induced apoptosis in LNCaP and PC-3 cells served as positive control. Fluorescence microscopy analyses revealed that CAP treated LNCaP and PC-3 cells exhibited striking changes in nuclear morphology with regard to the significantly decreased parameters nuclear area and perimeter, as well as a diminished major and minor nuclear axis compared to control cells and in accordance with the cycloheximide control (Fig 5). However, a disrounding of apoptotic nuclei calculated by the ratio of major and minor axis [34] could not be demonstrated. Despite similar effects of cycloheximide, CAP treatment exhibited more distinct changes in PC-3 cell morphology than in LNCaP cells. This is in accordance with higher annexin V specific signals in PC-3 cells as already mentioned above. Remarkably, we obtained further differential results by analysis of chromatin condensation. Accordingly to literature [35, 36], nuclei of CAP treated and DAPI stained LNCaP cells increased in brightness, whereas CAP treated PC-3 cells showed a decrease of nuclear DAPI signal. These observations could be evaluated by cycloheximide-induced apoptosis. In summary, we can only speculate about the underlying mechanisms, however, our data may reflect that differing cellular context could cause differential response to apoptotic stimuli.

Adding the data from three apoptosis assays detecting apoptotic events at different stages of apoptosis, thereby indicating different molecular effects, and based on different measuring procedures as well, our study provides strong evidence for apoptotic cell death following CAP treatment. This is in accordance with CAP-induced apoptosis of human peripheral blood mononuclear cells [37], as well as the induction of apoptotic factors in other solid cancer entities [5].

Despite the fact of growing knowledge about clinical implications the molecular insights behind the anti-proliferative and pro-apoptotic CAP effects are for the most unknown and matter of controversy. Physical cell damage by UV radiation or increased temperature was excluded by previous studies [7, 11]. More likely and in accordance with our results reactive species like ROS and RNS appear to be responsible for apoptotic cellular retrogression. Several investigators have reported different possible events triggered by intracellular reactive species including DNA damage [11], lipid peroxidation [38] or mitochondrial dysfunction [14, 39]. Here, it was possible to counteract CAP-mediated apoptosis by incubation with NAC, which is a synthetic precursor of intracellular cysteine and GSH [40]. This effect could not be observed by supplementing the cells with vitamin C, another cellular antioxidant, leading us to the assumption that CAP-driven anti-proliferation may rely specifically on the depletion of intracellular GSH. The necessity for sufficient GSH levels in fast proliferating cells has been demonstrated before. In fact, efforts have been made to decrease cellular GSH levels in tumor cells as pre-treatment for cancer therapy (summarized in: Markovic J et al. [41]). LNCaP as well as PC-3 cells seem to have low levels of total GSH (Fig 7) and may therefore be especially sensitive towards the treatment. In addition, CAP led to a rapid oxidation of the analysed 2-Cys Prxs, suggesting that an increase in hydrogen peroxide was induced by CAP treatment. This would not only affect the function of the Prxs, but also lead to a disruption or constitutive activation of redox signaling pathways. Prxs are overexpressed in various cancers and were suggested to function in the control of cell growth arrest/apoptosis and proliferation [16]. Bekeschus et al. have demonstrated that hydrogenperoxide assumes a dominant but not exculsive role in cellular oxidation of CAP treated blood cells [42]. Interestingly, we could show that CAP treatment affects the redox state of specific proteins in the cytosol. CAP treatment increased Prx1 oxidation state, whereas Prx2 did not show an increase in the catalytic, dimeric form. This is particularly interesting in terms of specificity of redox signaling events. Moreover, CAP treatment also affects mitochondria; the mitochondrial Prx3 was clearly more oxidized in terms of the dimeric form than in the argon treated control cells. It is important to mention that Prxs can be oxidized in different ways. For instance, Prxs were shown to be glutathionylated [43], but were also able to form a sulfenic acid within their catalytic mechanism that can be overoxidized to a sulfinic or sulfonic acid [44]. All these modifications cannot be detected with the 2-Cys redox blot used in this study, meaning that the monomeric band itself cannot be considered the reduced form.

Recently, CAP offered promising anti-neoplastic effects in other cancer entities such as pancreatic, colorectal and lung cancer as well as melanoma [5, 7, 8, 45–47]. These in vitro studies were already confirmed in vivo by nude mice models [48]. From our best knowledge this is the first report referring to CAP effects in PC. Surgical R0-resection is the most indispensable component in the curative therapy approach of PC. Thus, tumor-negative surgical margins may represent the most important variable for recurrence-free survival [49]. Local intraoperative CAP treatment as additional therapeutic intervention could prevent tumor recurrence and metastasis, as well as mollify surgical radicality and associated side effects. Moreover, endoscopic plasma application as demonstrated recently initiates manifold opportunities for innovative and less invasive anti-cancer treatment strategies [50]. CAP treatment of freely suspended cells as used in the experimental design of this study shows that CAP exerts a wide sphere of activity, which, to go a step further, may enable systemic applications. The feasibility of treating human tissue with CAP has already gained momentum [51]. Thereby, future research will clarify the substantial issue if CAP is capable to discriminate between physiological and malignant cells.
